# Does the Enigmatic *Wightia* Belong to Paulowniaceae (Lamiales)?

**DOI:** 10.3389/fpls.2019.00528

**Published:** 2019-04-30

**Authors:** Zhi Xia, Jun Wen, Zhiming Gao

**Affiliations:** ^1^College of Agronomy, Henan Agricultural University, Zhengzhou, China; ^2^Department of Botany, National Museum of Natural History, Smithsonian Institution, Washington, DC, United States

**Keywords:** *Wightia*, *Paulownia*, *Brandisia*, familial placement, phylogenetic relationship, Scrophulariaceae

## Abstract

The familial placement of *Wightia* has been controversial in the Lamiales, and the genus is currently placed in Paulowniaceae in APG IV. Phylogenetic analyses of *Wightia* and its close relatives in Lamiales are conducted using sequences of the complete chloroplast genomes as well as sequence data from nine chloroplast DNA regions (*atpB*, *matK*, *ndhF*, *psbBTNH*, *rbcL*, *rps4*, *rps16* intron, *trnL-F*, and *trnV-atpE*) and one mitochondrial gene *rps3*. The maximum likelihood and Bayesian analyses do not support a close relationship between *Wightia* and *Paulownia* of Paulowniaceae; instead the enigmatic *Wightia* is sister to Phrymaceae with strong support in all analyses. Hence *Wightia* should not be placed in Paulowniaceae. Because morphological data show *Wightia*’s affinity to both Phrymaceae and Paulowniaceae and prior nrITS data suggest its sister relationship to *Paulownia* of Paulowniaceae, it is likely that *Wightia* may have had a hybrid origin between early lineages of Phrymaceae and Paulowniaceae. It is therefore the best to exclude *Wightia* from Paulowniaceae and place the genus as unassigned until further nuclear data to test the hybrid hypothesis. The seven species of *Paulownia* constitute a monophyletic group, and Paulowniaceae is supported to be a monogeneric family, consistent with a series of morphological and floral development characters. The genus *Brandisia*, which was sometimes regarded as a close relative of *Wightia*, is supported to be nested within Orobanchaceae, as sister to *Pterygiella*. This sister relationship can be corroborated by fruit, seed and pollen morphological characters.

## Introduction

The familial placement of *Wightia* Wall. has been controversial. *Wightia* includes two species distributed mainly in Burma, India, Malaysia, Nepal, Vietnam, and Yunnan of China (van Steenis, 1949; Maheshwari, 1961; Hong et al., 1998). It was initially placed in Bignoniaceae based on the characters such as seeds that are winged but without endosperm and extra-floral nectarines under the leaf surface (Wallich, 1830; Hallier, 1903; Campbell, 1930; Li, 1947; Lawrence, 1951; Maheshwari, 1961). It was also included in Scrophulariaceae sensu lato on the basis of its two locular ovaries, and fruit dehiscing characters (Bentham and Hooker, 1876; Pennell, 1920; van Steenis, 1949; Willis, 1955; Hu, 1959; Hong et al., 1998). Recently Fischer (2004) mentioned that *Wightia*, together with *Brandisia* Hook. f. & Thomson, constituted the tribe Wightieae of Paulowniaceae of Nakai (1949). Stevens (2001) onward and APG IV (2016) placed *Wightia* and *Paulownia* Siebold & Zucc. as members of Paulowniaceae.

*Paulownia* consists of seven species native to eastern Asia and it is widely distributed in China and cultivated worldwide (Hong et al., 1998). The genus is well-known for its economic importance as the sources for rapidly growing timbers (Hu, 1959). The woody genus *Paulownia* was usually placed in Scrophulariaceae s.l. (Bentham and Hooker, 1876; Wettstein, 1891; Takhtajan, 1997; Hong et al., 1998), but it was sometimes positioned between Scrophulariaceae s.l. and Bignoniaceae (Campbell, 1930; Armstrong, 1985) or treated as a member of Bignoniaceae (Hallier, 1903; Takhtajan, 1980; Cronquist, 1981), although Nakai (1949) treated it as its own family, Paulowniaceae.

*Brandisia* comprises eleven species which are distributed in subtropical eastern Asia (Hong et al., 1998). The genus was placed in the tribe Cheloneae (Bentham and Hooker, 1876) or together with *Wightia* and *Paulownia* in Paulownieae (Tsoong, 1979) in Scrophulariaceae s.l., but some workers speculated that it may belong to other families, such as Loganiaceae, Solanaceae, Bignoniaceae, Pedaliaceae, Myoporaceae, and Verbenaceae (Campbell, 1930; Li, 1944, 1947).

Molecular phylogenetic analyses have revealed that the traditionally circumscribed Scrophulariaceae s.l. is polyphyletic (Olmstead and Reeves, 1995; Olmstead et al., 2001; Oxelman et al., 2005; Tank et al., 2006). These studies have resulted in circumscriptions and new descriptions of families to encompass the monophyletic lineages that were recovered in Lamiales. *Paulownia* was tentatively elevated to the monogeneric family Paulowniaceae (Beardsley and Olmstead, 2002), but its monophyly has not been tested because previous phylogenetic studies often sampled only *P. tomentosa*. Based on molecular data [*rps16* intron, *trnL-F* region and nuclear ribosomal internal transcribed spacer (nrITS)] and chemical evidence, Zhou et al. (2014) argued that *Wightia* is closely allied to Paulowniaceae. However, there was phylogenetic conflict between nrITS and plastid loci (*rps16* intron and *trnL-F* region) concerning the position of *Wightia*. *Wightia* was sister to *Paulownia* in the ITS tree, but it formed a clade with Phrymaceae in the chloroplast (combined *trnL-F and rps16*) tree. Zhou et al. (2014) also found that *Brandisia* (sampling only one species *Brandisia swinglei*) is not closely related to *Wightia*, instead the latter is nested within Orobanchaceae. Other molecular systematic studies (Oxelman et al., 2005; Bennett and Mathews, 2006; McNeal et al., 2013) also showed *Brandisia* (sampling only one species *B. hancei*) as a member of Orobanchaceae. However, the accurate systematic position of *Brandisia* within Orobanchaceae is uncertain. *Brandisia* was sister to the clade [Rhinantheae (Pedicularideae, Buchnereae)] in the nuclear *PHYA* gene topology with moderate support (BS = 72; 75, ML support) (Bennett and Mathews, 2006; McNeal et al., 2013) and in the nrITS tree (PP = 0.82 only) with weak support (Yu et al., 2018). However, it was sister to *Pterygiella* in nrITS and plastid (*matK* and *rps2*) trees both with low support (McNeal et al., 2013), or in the combined plastid (*matk, rbcL*, *rps2*, *rps16*, *trnK-matK*, and *trnH-psbA*) tree with moderate support (BS = 69, ML support; PP = 0.96) (Yu et al., 2018). The topological conflict between McNeal et al. (2013) and Yu et al. (2018) results with nrITS may be attributed to the difference in taxon sampling in these studies. With the majority of *Brandisia* species not included in the previous molecular phylogenetic studies, McNeal et al. (2013) suggested that further work on the genus is desirable because it occupies a pivotal place in the phylogeny, as the likely sister of the clade [Rhinantheae (Pedicularideae, Buchnereae)], which contains most species diversity in Orobanchaceae.

It is necessary to increase the sampling of key putative relatives of *Wightia* and include additional DNA characters in order to accurately determine the familial placement of *Wightia* in Lamiales. In recent years, the chloroplast genomes have been widely used to resolve difficult phylogenetic relationships in plants (e.g., Jansen et al., 2007; Zhang et al., 2015; Xu et al., 2017; Wen et al., 2018). This study is conducted with a comprehensive sampling of putative relatives of *Wightia* in Lamiales s.l. and using nine chloroplast DNA regions (*atpB, matK, ndhF, psbBTNH, rbcL, rps4, rps16* intron*, trnL-F*, *and trnV-atpE*) and one mitochondrial gene *rps3* that have been shown to be particularly informative in the Lamiales s.l. (Refulio-Rodriguez and Olmstead, 2014). We further conduct a second phylogenetic analysis using a selected sampling of the putative relatives of *Wightia* in Lamiales based on chloroplast genome sequences. The complete chloroplast (cp) genome sequences of *Wightia*, *Brandisia*, *Mazus*, and *Phryma* are herein reported for the first time. The goals of this study are to (1) test the familial placement of *Wightia*, and (2) determine the taxonomic composition of Paulowniaceae by broadly sampling species of *Paulownia*, *Brandisia*, as well as Phrymaceae, Mazaceae, and Orobanchaceae in Lamiales.

## Materials and Methods

### Taxon Sampling

The taxon sampling consisted of 110 samples representing all the families of Lamiales. One species of *Wightia*, all seven species of *Paulownia* and six species of *Brandisia* were sampled for the first time in this study. In addition, data of the remaining samples in this study are mostly from McNeal et al. (2013), Refulio-Rodriguez and Olmstead (2014), and Yu et al. (2018). We also selected 22 representative genera of nine major clades in Orobanchaceae (McNeal et al., 2013; Yu et al., 2018) to test the placement of *Brandisia*. Two species: *Solanum lycopersicum* L. (Solanaceae) and *Gelsemium sempervirens* (L.) J.St.-Hil. (Gelseminaceae) were selected as outgroups based on Refulio-Rodriguez and Olmstead (2014). The chloroplast genome data of four species (including *Wightia speciosissima*, *B. swinglei*, *Mazus pumilus*, and *Phryma leptostachya* subsp. *asiatica*) were reported for the first time in this study. We selected one species of *Wightia*, two species of *Paulownia* (Paulowniaceae), two genera of Gesneriaceae, four genera of Plantaginaceae, one genus of Scrophulariaceae sensu stricto, two genera of Phrymaceae, two genera of Mazaceae, 16 representative genera of eight major clades (McNeal et al., 2013; Yu et al., 2018) in Orobanchaceae, two genera of Acanthaceae, three genera of Bignoniaceae, five genera of Lamiaceae, and one genus of Pedaliaceae, one genus of Verbenaceae, and two genera of Lentibulariaceae. *Solanum bulbocastanum* Dunal and *Hyoscyamus niger* L. of Solanaceae were selected as outgroups. Voucher specimens are deposited in the Herbarium of Henan Agricultural University (HEAC). Voucher information and GenBank accession numbers of the sequences used in this study are provided in Supplementary Tables S1, S2.

### DNA Extraction, PCR Amplification, and Sequencing

Total genomic DNA was extracted from leaf tissue samples preserved in silica gel or leaves removed from herbarium specimens using the modified 2 × CTAB method (Doyle and Doyle, 1987) and the Plant Genomic DNA Kit (DP305) from Tiangen Biotech (Beijing) Co., Ltd., China. We sequenced nine plastid regions, including six coding regions (*atpB*, *matK*, *ndhF*, *psbBTNH*, *rbcL*, and *rps4*), three noncoding regions (*rps16* intron, *trnL-F* intron and spacer, and *trnV-atpE* spacer), and one mitochondrial coding region (*rps3*). The primer information of PCR amplification and amplification reactions for all chloroplast and the mitochondrial genes were as in Refulio-Rodriguez and Olmstead (2014). We included available DNA sequences of the above mentioned gene regions from GenBank.

Amplification reactions for all ten genes were run according to the following steps: (1) a denaturing step at 94°C for 45 s, (2) 35 cycles with a denaturing step at 94°C for 45 s, and an annealing step at 52°C for 45 s, an extension step at 72°C for 90 s, and (3) a final extension at 72°C for 10 min. Missing sequences are a consequence of amplification failure or lack of DNA availability. PCR products were purified with a PCR purification kit (UNIQ-10, Sangon, Shanghai, China). Sequencing primers were the same as amplification primers. Sequencing was performed on an ABI 3730xl DNA sequencer (Applied Biosystems) by Sunbiotech Co., Ltd., Beijing.

### Chloroplast Genome Sequencing, Assembly, and Annotation

DNA samples were randomly fragmented into 400–600 bp fragments using an ultrasonicator. An Illumina paired-end DNA library with 500-bp insert size was constructed using a NEBNext^®^ UltraTM DNA Library Prep Kit following the manufacturer’s instructions. Paired-end sequencing (2 × 150 bp) was conducted on an Illumina HiSeq X platform.

The paired-end reads were qualitatively assessed and initially assembled with SPAdes 3.6.1 (Bankevich et al., 2012), using k-mer ranging from 57 to 99. Contigs of low sequencing depths were discarded. The remaining contigs may contain the information not only from the chloroplast genome but also from the nuclear genome and the mitochondrial genome. Next, chloroplast genome sequence contigs were selected from SPAdes software by performing a BLAST search using the *Triaenophora shennongjiaensis* X. D. Li, Y. Y. Zan & J. Q. Li chloroplast genome sequence as a reference (GenBank accession number: MH071405) (Xia and Wen, 2018). The selected contigs were further assembled with Sequencher 5.4.5 (Gene Codes, Ann Arbor, MI, United States). Small gaps in the assemblies were bridged with specific primers designed for PCR based on their flanking sequences and then by Sanger sequencing. Based on the reference chloroplast genome, the four junctions between the inverted repeats (IRs) and single copy regions were checked by amplification with specific primers followed by Sanger sequencing (Dong W. et al., 2013). Chloroplast genome annotation was performed with Plann (Huang and Cronk, 2015) using the *T. shennongjiaensis* reference sequence from GenBank. The annotated GenBank files were used to construct the circular plastid genome maps with the online program Organellar Genome DRAW (OGDRAW) (Lohse et al., 2013) and then the annotated cp genome sequences were submitted to GenBank with the accession number MK381318 (*W. speciosissima*), MK381315 (*B. swinglei*), MK381316 (*M. pumilus*), and MK381317 (*P. leptostachya* subsp. *asiatica*).

### Sequence Alignment and Phylogenetic Analysis

Initial automated alignments of the individual genes were made using the MAFFT (Katoh and Standley, 2013) with the E-INS-I algorithm in Geneious. The data from nine chloroplast regions, and the mitochondrial gene *rps3* were analyzed separately. The chloroplast genome sequences were performed based on the all common protein coding genes (PCGs) (*Ycf1* gene was excluded because of high diversity in Lamiales). Gaps were treated as missing data. The data matrix combining all 10 genes, and combining PCGs of chloroplast genome were performed by using both maximum likelihood (ML) and Bayesian inference (BI) methods.

The ML analyses were conducted using RAxML (version 8.2; Stamatakis, 2014). These analyses used the GTR substitution model with gamma-distributed rate heterogeneity among sites and the proportion of invariable sites estimated from the data. The concatenated plastid dataset was partitioned by gene. Support values for the node and clade were estimated from 1000 bootstrap replicates. BI analyses were performed using MrBayes vers. 3.2.6 (Ronquist et al., 2012). The Markovchain Monte Carlo (MCMC) analysis was run in MrBayes for 10, 000, 000 generations for each dataset. We checked for stationarity in Tracer version1.4 (Drummond and Rambaut, 2007) by confirming an ESS of greater than 200 for all parameters and by visually inspecting the distributions of the sampled states. The first 25% of samples were discarded as burn-in (Huelsenbeck and Ronquist, 2001) and the remaining trees were used to generate a majority-rule consensus tree. The ML tree and BI tree were visualized using FigTree version 1.4.2.

## Results

### Chloroplast Genome Features

Using an Illumina HiSeq X System, samples of the four taxa: *W. speciosissima*, *B. swinglei*, *M. pumilus* and *P. leptostachya* subsp. *asiatica*, were sequenced via the genome skimming approach (Zhang et al., 2015; Zimmer and Wen, 2015), producing 26, 779, 390; 29, 029, 530; 39, 331, 112; and 7, 460, 482 paired end raw reads (150 bp average read lengths).

**FIGURE 1:**
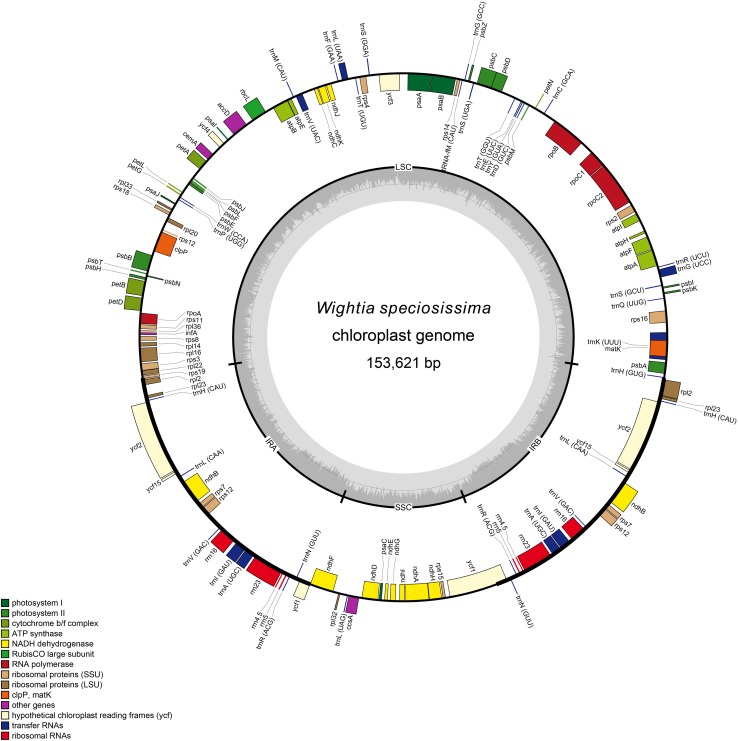
Gene map of the chloroplast genome of *Wightia speciosissima*.

The chloroplast genomes of *W. speciosissima*, *B. swinglei*, *M. pumilus* and *P. leptostachya* subsp. *asiatica* had a total sequence length of 153, 621 bp, 155, 344 bp, 153, 034 bp, and 153, 167 bp, respectively. The chloroplast genomes showed a typical quadripartite structure, consisting of a pair of IRs (25, 797 bp, 26, 498 bp, 25, 872 bp, and 25,456 bp, respectively) separated by the LSC (84, 393 bp, 84, 650 bp, 83, 839 bp, and 84,877 bp, respectively) and SSC (17, 634 bp, 17, 698 bp, 17, 451 bp, and 17, 378 bp, respectively) regions. For the four chloroplast genomes (*W. speciosissima*, *B. swinglei*, *M. pumilus* and *P. leptostachya* subsp. *asiatica*), the average GC content was 37.7, 38.1, 37.8, and 37.7%, respectively (Table 1). The annotated chloroplast genomes of four species were represented in four circular maps (Figure 1 and Supplementary Figures S1–S3). The chloroplast genomes of *W. speciosissima* and *B. swinglei* harbored 112 different genes, including 78 protein-coding genes, 30 tRNA genes and 4 rRNA genes. The chloroplast genome of *M. pumilus* harbored 114 different genes, including 81 protein-coding genes, 29 tRNA genes, and 4 rRNA genes. The chloroplast genome of *P. leptostachya* subsp. *asiatica* had 112 different genes, including 79 protein-coding genes, 29 tRNA genes, and 4 rRNA genes.

**TABLE 1:** Summary of complete chloroplast genome features of the four species.

**Name of**	***Wightia***	***Brandisia***	***Mazus***	***Phryma***
**taxon**	***speciosissima***	***swinglei***	***pumilus***	***eptostachya***
				**ssp. *asiatica***
Genome length (bp)	153, 621	155, 344	153,034	153, 167
LSC length (bp)	84, 393	84, 650	83, 839	84, 877
IR length (bp)	25, 797	26, 498	25, 872	25, 456
SSC length (bp)	17, 634	17, 698	17, 451	17, 378
Total gene number	112	112	114	112
No. of protein coding genes	78	78	81	79
No. rRNA genes	30	30	29	29
No. tRNA genes	4	4	4	4
GC content in genome (%)	37.7%	38.1%	37.8%	37.7%

### Phylogenetic Analyses Using Nine Chloroplast Regions and One Mitochondrial Gene

**FIGURE 2:**
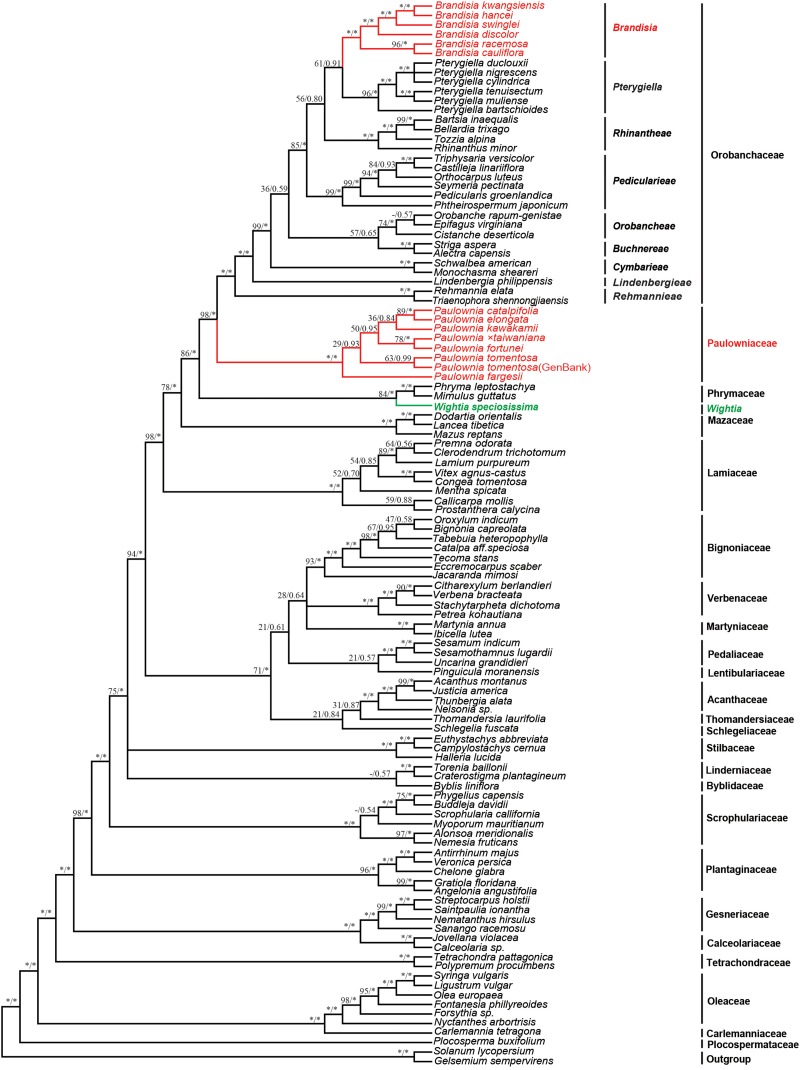
Bayesian tree based on the combined nine plastid markers (*atpB, matK, ndhF, psbBTNH, rbcL, rps4, rps16* intron*, trnL-F, and trnV-atpE*). Number above branches are ML bootstrap values/Bayesian posterior. An asterisk indicates bootstrap value of 100 or posterior probability of 1.00; a hyphen (-) indicates the branch was not obtained in the ML bootstrap consensus.

The 9-marker chloroplast regions combined data matrix consists of 14,789 bp in aligned length. The length of alignment, number of parsimony informative characters and PIC percentage (%) of each gene were shown in Supplementary Table S3. The chloroplast sequence data provided good resolution in the phylogenetic analyses overall. The topologies based on ML and Bayesian methods were both highly supported (Figure 2). *Wightia* is sister to Phrymaceae [Bootstrap (BS) = 84; posterior probability (PP) = 1.00]. In Orobanchaceae, nine clades (including *Brandisia* group and *Pterygiella* group) are well resolved (PP = 1.00). Orobanchaceae is sister to Paulowniaceae (BS = 98; PP = 1.00), and the Orobanchaceae – Paulowniaceae clade is sister to Phrymaceae (BS = 86; PP = 1.00), with the Orobanchaceae – Paulowniaceae – Phrymaceae clade then sister to Mazaceae (BS = 78; PP = 1.00). All seven species in *Paulownia* constitute monophyletic group with the maximum support (BS = 100; PP = 1.00). The six sampled species of *Brandisia* form one clade (BS = 100; PP = 1.00) that is nested within Orobanchaceae. *Brandisia* is sister to *Pterygiella* (BS = 61; PP = 0.91), and the *Brandisia – Pterygiella* clade is sister to Rhinantheae (BS = 56; PP = 0.80). Within *Paulownia*, *P. fortune* is sister to *Paulownia × taiwaniana* with BS = 78 and PP = 1.00, and *P. elongata* is sister to *P. catalpifolia* with BS = 89 and PP = 1.00.

The mitochondria gene *rps3* data matrix consists of 1561 bp in aligned length. The length of alignment, number of parsimony informative characters and PIC percentage (%) of mitochondria gene *rps3* were showed in Supplementary Table S3. The topologies based on ML and Bayesian methods showed in Supplementary Figure S4. *Wightia* is sister to one clade including Phrymaceae and Mazaceae (BS = 65; PP = 0.95). Paulowniaceae is sister to *Wightia* – Phrymaceae-Mazaceae clade (PP = 0.89), and then sister to Orobanchaceae (BS = 92; PP = 1.00). All seven species in *Paulownia* constitute monophyletic group with the maximum support (BS = 99; PP = 1.00). The six sampled species of *Brandisia* form one clade (BS = 62; PP = 0.97).

### Phylogenetic Analyses Using Chloroplast Genome Sequences

The 79 protein-coding plastid genes sequence (PCGs) from the chloroplast genome of *Wightia* and its related taxa in the Lamiales were shown in the Supplementary Table S4. The data set from PCGs of the whole chloroplast genome provided the best resolution in the phylogenetic analyses with high bootstrap support values. The topologies based on the ML and BI methods were highly supported and congruent. *Wightia* is sister to Phrymaceae (BS = 100; PP = 1.00). The two sampled species of *Paulownia* form a clade with the maximum support (BS = 100; PP = 1.00). *Brandisia* is included in Orobanchaceae, which is sister to Rhinantheae (BS = 94; PP = 1.00). *Paulownia* is sister to Orobanchaceae with BS = 99 and PP = 1.00. Orobanchaceae and Paulowniaceae (*Paulownia* only) are then sister to Phrymaceae with high support (BS = 99; PP = 1.00). Mazaceae is sister to Lamiaceae with moderate support (BS = 65; PP = 0.95).

## Discussion

### On the Familial Placement of *Wightia*

**FIGURE 3:**
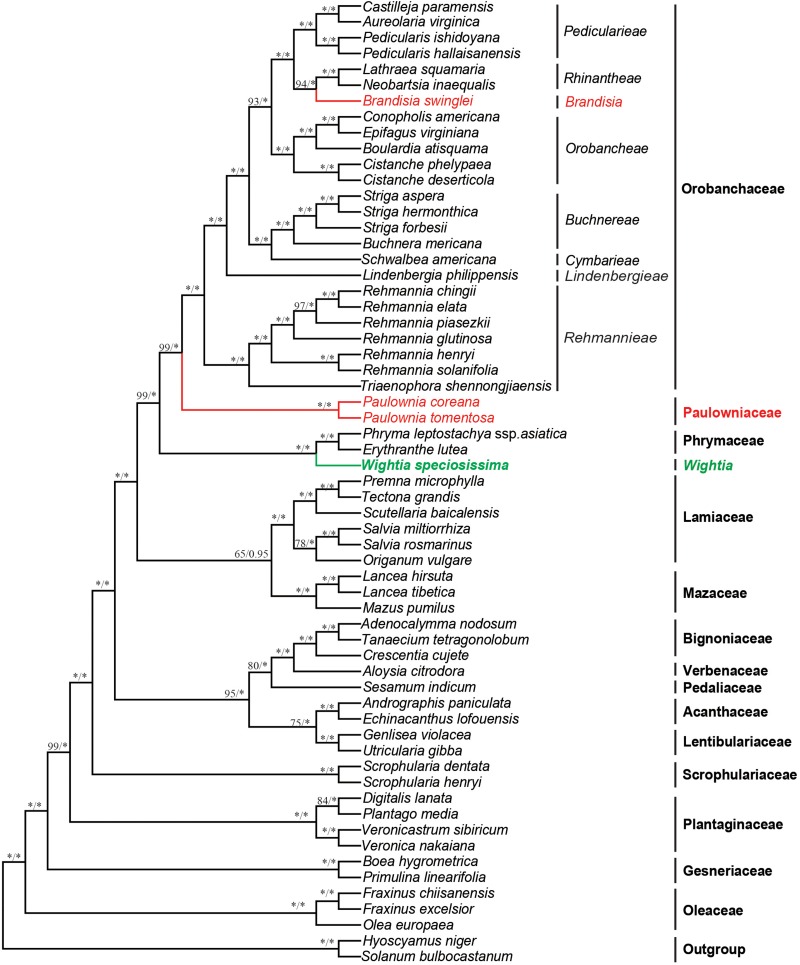
Bayesian tree inferred from the sequence of the protein coding genes (PCGs) of the chloroplast genome data. Numbers above branches are ML bootstrap values/Bayesian posterior probabilities. An asterisk indicates bootstrap value of 100 or posterior probability of 1.00.

The analyses based on DNA sequences from the 10 combined chloroplast and mitochondrial regions and the complete chloroplast genome data do not support a clade of *Wightia* and *Paulownia*. Even though *Wightia* was placed variously in Scrophulariaceae s.l., Bignoniaceae or Paulowniaceae in Lamiales (Wallich, 1830; Bentham and Hooker, 1876; Hallier, 1903; Pennell, 1920; Campbell, 1930; Li, 1947; van Steenis, 1949; Lawrence, 1951; Willis, 1955; Hu, 1959; Maheshwari, 1961; Hong et al., 1998; Fischer, 2004), our results support *Wightia* as sister to Phrymaceae (BS = 84,100; PP = 1.00,1.00; Figures 2, 3) or sister to the Phrymaceae - Mazaceae clade (BS = 65; PP = 0.95; Supplementary Figure S4). The *Wightia*-Phrymaceae clade is then sister to the large clade that includes Orobanchaceae and Paulowniaceae with high support (Figures 2, 3).

The sister relationship between *Wightia* and Phrymaceae was initially reported by Zhou et al. (2014) using chloroplast *trnL-F* region and *rps16* intron. Because their nrITS tree showed *Wightia* as sister to *Paulownia* with moderate support, the authors placed *Wightia* in Paulowniaceae (Zhou et al., 2014). The placement of *Wightia* in Paulowniaceae was followed by Stevens (2001 onward) and APG IV (2016). Our results clearly show that *Wightia* should not be simply placed in Paulowniaceae. *Wightia* species are hemiepiphytic and evergreen lianas which are distinctively different from taxa of other families in Lamiales (Fischer, 2004). *Wightia* and Phrymaceae share a series of morphological characters, such as two lateral bracteoles at the base of the pedicel just above the subtending bract, and capsules oblong-ovoid or narrowly ellipsoid (Hong et al., 1998; Fischer, 2004). In addition, the pollen of *Wightia* also shares the type IIb character of pollen in *Mimulus* of Phrymaceae in the following respects: trocolporate, exine microreticulate, and mean polar axis 25–35 μm (Argue, 1980; Wei, 1989). These morphological characters thus support a close relationship between *Wightia* and Phrymaceae. However, several other morphological characters and chemical constituents also suggest a possible close relationship between *Wightia* and *Paulownia*. They both bear large, entire, opposite leaves, lateral or terminal thyrses, thick and smooth calyx tubes, and two-valved capsules with many winged seeds (Maheshwari, 1961; Zhou et al., 2014). In light of the morphological evidence that shows that *Wightia* shares important characters with both Phrymaceae and Paulowniaceae, we propose that the incongruent position of *Wightia* based on nrITS and chloroplast sequences (c.f., Zhou et al., 2014; this study) might be caused by a likely hybrid origin of *Wightia* involving early lineages associated with Phrymaceae and Paulowniaceae. Further studies by sampling both species of *Wightia* and utilizing more nuclear sequences are needed as the next step to better understand the evolutionary history and the taxonomic position of *Wightia*, especially testing its hybrid origin hypothesis. If the hybrid origin is confirmed, it is the best to recognize *Wightia* as a distinct family Wightiaceae. At present, we suggest treating *Wightia* conservatively as unassigned at the familial level until further evidence from the nuclear genome. It is likely that *Wightia* may need to be placed in its own family.

Our results support recognizing Paulowniaceae as the monogeneric family (Nakai, 1949; Beardsley and Olmstead, 2002; Erbar and Gülden, 2011). The monogeneric Paulowniaceae possesses a series of diagnostic features which distinguish it from other families in Lamiales, i.e., tree habit, woody fruit, the unidirectional initiation of calyx, and corolla lobes from the adaxial to the abaxial side, late sympetaly petal but ledges connecting the petal arise relatively early, tubular stigma with papillae inside a dilated chamber (Erbar and Gülden, 2011) and the plain surface of the placenta with distinct long and slightly angular structures (Rebernig and Weber, 2007). Erbar and Gülden (2011) noted that *Paulownia tomentosa* shows the unidirectional initiation of calyx and corolla lobes from the adaxial to the abaxial side and plain surface of the placenta with distinct long and slightly angular structures, which are unusual in Lamiales. Hence they argued for the isolated position of the monogeneric family Paulowniaceae.

The position of Mazaceae (Reveal, 2011) still needs to be further explored. Mazaceae is sister to the clade containing Orobanchaceae, Paulowniaceae, and Phrymaceae in the tree based on nine chloroplast markers (Figure 2), consistent with Xia et al. (2009), Schäferhoff et al. (2010), and Refulio-Rodriguez and Olmstead (2014). But in the tree based on complete chloroplast genome, Mazaceae is sister to Lamiaceae with bootstrap support BS = 65 and PP = 0.95. This inconsistence may be caused by the limited sampling of Mazaceae and Phrymaceae in the chloroplast genome data set. Nevertheless, the Mazaceae position as sister to the clade of Orobachaceae, Paulowniaceae, and Phrymaceae had only 50% ML bootstrap support and 0.62 PP in Schäferhoff et al. (2010), and 34% MP bootstrap support in Refulio-Rodriguez and Olmstead (2014). The systematic position of Mazaceae deserves further test with greater taxon sampling in Mazaceae and its putative relatives (Phrymaceae) and by using chloroplast genomes and more nuclear markers.

### Species Relationships Within Paulowniaceae

Within the monogeneric Paulowniaceae, our results shed some insights into the evolution of the genus *Paulownia*, as we sampled all seven species of the genus. *Paulownia fortunei* is shown to be sister to *P × taiwaniana* with high support (Figure 2). *Paulownia × taiwaniana* was reported as a natural hybrid species between *P. fortunei* and *P. kawakamii* with the latter as the maternal parent based on RAPD markers, chloroplast RFLP data (using one restriction enzyme only) and morphology (Lin and Wang, 1991; Wang et al., 1994). However, *P. kawakamii* as the maternal parent of *P × taiwaniana* is not supported by our results. Our results suggest *P. fortunei* as the likely maternal parent of *P × taiwaniana*, because the two species are sisters in the tree based on the maternally inherited chloroplast data (Figure 2). Furthermore, *Paulownia elongata* is sister to *P. catalpifolia* with high support. The close relationship between *P. elongata* and *P. catalpifolia* is also supported morphologically by their shared pubescent capsules, and calyx lobes shorter than tube (Hu, 1959; Hong et al., 1998). Our present study is the first to confirm the monophyly of *Paulownia* with all species sampled. But our analysis is limited to chloroplast and mitochondrial data. Fast-evolving, biparently inherited nuclear markers are needed to disentangle the species relationships of *Paulownia* and test potential hybrid speciation in the genus (Wang et al., 1994), using dense sampling of populations in the genus.

### On the Position of *Brandisia* Within Orobanchaceae

Our study sampled six species of *Brandisia*, and shows that the genus is nested within Orobanchaceae. Inclusion of *Brandisia* within Orobanchaceae is consistent with the result of recent phylogenetic studies (Oxelman et al., 2005; Bennett and Mathews, 2006; McNeal et al., 2013; Yu et al., 2018). *Brandisia* as part of Orobanchaceae is supported by its capsules having half or partly exserted from the persistent calyx tubes and its hemiparasitic habits (Chin, 1979; Zhang, 1990; Hong et al., 1998; Xia et al., 2009). Yet the phylogenetic position of *Brandisia* within Orobanchaceae has been controversial in previous studies (Oxelman et al., 2005; Bennett and Mathews, 2006; McNeal et al., 2013; Yu et al., 2018). By increasing the sampling of *Brandisia*, the phylogenetic tree (Figure 2) using nine chloroplast gene regions showed that *Brandisia* is sister to *Pterygiella* in Orobanchaceae (BS = 61; PP = 0.91) which is consistent with the result of Yu et al. (2018), and Rhinantheae is sister to the clade containing *Brandisia* and *Pterygiella* in Orobanchaceae (BS = 56; PP = 0.80). Without sampling *Pterygiella*, our analyses using complete chloroplast genome data placed *Brandisia* as sister to Rhinantheae in Orobanchaceae with BS = 94 and PP = 1.00 (Figure 3). Fruit and seed characters also showed close relationships between *Brandisia* and *Pterygiella*, as both have eglandular hairs on the surface of capsules and reticulate seeds (Dong L.-N. et al., 2013; Dong et al., 2015). Furthermore, the pollen grains of *Brandisia*, *Pterygiella* and most genera in Rhinantheae are commonly tricolpate, medium-sized, circular or subcircular, and having long and acute-ended colpi with a granulate membrane, supported the close relationships among them (Wei, 1989; Lu et al., 2007).

The six species we sampled in *Brandisia* formed a monophyletic group with maximum support in the combined chloroplast and mitochondrial gene tree (Figure 2). *Brandisia* has a series of morphological characters which are distinctive from other genera in Orobanchaceae, such as infundibular corollas with strongly reflexed corolla lobes, and anthers with dense hairs (Ren et al., 2018). Ren et al. (2018) reported that the anther hairs function as a secondary pollen presentation mechanism and play a key role in restricting pollen loss after anther dehiscence, hence facilitating reproductive fitness in delayed selfing in *Brandisia hancei*. The secondary pollen presentation on anthers hairs is unique and may turn out to be a synapomorphy of *Brandisia*.

In conclusion, our results argue that *Wightia* should be removed from Paulowniaceae. As *Wightia* may be of hybrid origin between early lineages of Phrymaceae and Paulowniaceae, we suggest treating *Wightia* conservatively as unassigned at the familial level. If its hybrid origin is confirmed with further nuclear data, *Wightia* may need to be recognized as its own family. The systematic position of Mazaceae deserves further studies. *Brandisia* is sister to *Pterygiella* in Orobanchaceae, whichis corroborated by fruit and seed characters, pollen morphology and molecular data.

## Author Contributions

ZX and JW conceived the study and interpreted the results. ZX and ZG collected and analyzed the data.

## Conflict of Interest Statement

The authors declare that the research was conducted in the absence of any commercial or financial relationships that could be construed as a potential conflict of interest.

## Supplementary Material

FIGURE S1 Gene map of the chloroplast genome of *Brandisia swinglei*.

FIGURE S2 Gene map of the chloroplast genome of *Mazus pumilus.*

FIGURE S3 Gene map of the chloroplast genome of *Phryma leptostachya* subsp. *asiatica*.

FIGURE S4 Bayesian tree inferred from the sequence of one mitochondrial gene *rps3* data. ML bootstrap values (>50%)/Bayesian posterior probabilities (>0.50) are shown above branches. An asterisk indicates bootstrap value of 100 or posterior probability of 1.00; a hyphen (-) indicates the branch was not obtained in the ML bootstrap consensus.

TABLE S1 Species included in this study, with assignment to order (where appropriate), family, origin and voucher information (herbarium acronym), and GenBank accession numbers (chloroplast *atpB*, *matK*, *ndhF*, *psbBTNH*, *rbcL*, *rps4*, *rps16* intron, *trnL-F*, and *trnV-atpE*, and mitochondrial *rps3*). “na” indicates no sequence available for this region for the accession.

TABLE S2 Voucher information and GenBank Accessions of chloroplast genomes used in this study.

TABLE S3 The nine chloroplast DNA regions and one mitochondrial gene used in the phylogenetic analyses of Lamiales.

TABLE S4 The 79 protein-coding plastid genes used in the phylogenetic analyses of Lamiales.

## References

[B1] Apg I. V. (2016). An update of the angiosperm phylogeny group classification for the orders and families of flowering plants: APG IV. *Bot. J. Linn. Soc.* 181 1–20.

[B2] Argue C. L. (1980). Pollen morphology in the genus *Mimulus* (Scrophulariaceae) and its taxonomic significance. *Am. J. Bot.* 67 68–87.

[B3] Armstrong J. E. (1985). The delimitation of Bignoniaceae and Scrophulariaceae based on floral anatomy and the placement of problem genera. *Am. J. Bot.* 72 755–766.

[B4] Bankevich A. Nurk S. Antipov D. Gurevich A. A. Dvorkin M. Kulikov A. S. (2012). SPAdes: a new genome assembly algorithm and its applications to single-cell sequencing. *J. Comput. Biol.* 19 455–477. 10.1089/cmb.2012.0021 22506599 PMC3342519

[B5] Beardsley P. M. Olmstead R. G. (2002). Redefining Phrymaceae: the placement of *Mimulus*, tribe Mimuleae, and *Phryma*. *Am. J. Bot.* 89 1093–1102. 10.3732/ajb.89.7.1093 21665709

[B6] Bennett J. Mathews S. (2006). Phylogeny of the parasitic plant family Orobanchaceae inferred from Phytochrome A. *Am. J. Bot.* 93 1039–1051. 10.3732/ajb.93.7.1039 21642169

[B7] Bentham G. Hooker J. D. (1876). “Scrophulariaceae,” in *Genera Plantarum*, Vol. 2 eds Bentham G. Hooker J. D. (London: Williams and Norgaate), 913–980.

[B8] Campbell D. H. (1930). The relationships of *Paulownia*. *Bull. Torrey Bot. Club.* 57 47–50.

[B9] Chin T.-L (1979). “*Rehmannia* and *Triaenophora*,” in *Flora Reipublicae Popularis Sinicae*, *Part 2 Scrophulariaceae*, Vol. 67 eds Tsoong P. C. Yang H. P. (Beijing: Science Press), 212–222.

[B10] Cronquist A. (1981). *An Integrated System of Classification of Flowering Plants.* New York, NY: Columbia University Press.

[B11] Dong L.-N. Wang H. Wortley A. H. Li D.-Z. Lu L. (2015). Fruit and seed morphology in some representative genera of tribe Rhinantheae sensu lato (Orobanchaceae) and related taxa. *Plant Syst. Evol.* 301 479–500.

[B12] Dong L.-N. Wang H. Wortley A. H. Lu L. Li D.-Z. (2013). Phylogenetic relationships in the *Pterygiella* complex (Orobanchaceae) inferred from molecular and morphological evidence. *Bot. J. Linn. Soc.* 171 491–507.

[B13] Dong W. Xu C. Cheng T. Lin K. Zhou S. L. (2013). Sequencing angiosperm plastid genomes made easy: a complete set of universal primers and a case study on the phylogeny of Saxifragales. *Genome Biol. Evol.* 5 989–997. 10.1093/gbe/evt063 23595020 PMC3673619

[B14] Doyle J. J. Doyle J. L. (1987). A rapid DNA isolation procedure for small quantities of fresh leaf tissue. *Phytochem. Bull.* 19 11–15.

[B15] Drummond A. J. Rambaut A. (2007). BEAST: bayesian evolutionary analysis by sampling trees. *BMC Evol. Biol.* 7:214. 10.1186/1471-2148-7-214 17996036 PMC2247476

[B16] Erbar C. Gülden C. (2011). Ontogeny of the flowers in *Paulownia tomentosa*-A contribution to the recognition of the resurrected monogeneric family Paulowniaceae. *Flora* 206 205–218.

[B17] Fischer E. (2004). “Scrophulariaceae,” in *The Families and Genera of Vascular Plants. Vol. 7. Flowering Plants, Dicotyledons: Lamiales (Except Acanthaceae Including Avicenniaceae)*, ed. Kaderei J. W. (Berlin: Springer).

[B18] Hallier H. (1903). Ueber die abgrenzung und verwandtschaft der einzelnen sippen bei den scrophularineen. *Bull. Herb. Boiss.* 3 181–207.

[B19] Hong D.-Y. Yang H.-B. Jin C.-L. Holmgren N. H. (1998). “Scrophulariaceae,” in *Flora of China Editorial Committee*, Vol. 18 eds Wu Z. Y. Raven P. H. (Beijing: Missouri Botanical Garden Press).

[B20] Hu S.-Y. (1959). A monograph of the genus *Paulownia*. *Quart. J. Taiwan Mus.* 12 1–54.

[B21] Huang D. I. Cronk Q. C. B. (2015). Plann: a command-line application for annotating plastome sequences. *Appl. Plant Sci.* 3 as.1500026. 10.3732/apps.1500026 26312193 PMC4542940

[B22] Huelsenbeck J. P. Ronquist F. (2001). MRBAYES: bayesian inference of phylogenetic trees. *Bioinformatics* 17 754–755. 11524383 10.1093/bioinformatics/17.8.754

[B23] Jansen R. K. Cai Z. Raubeson L. A. Daniell H. Leebens-Mack J. Müller K. F. (2007). Analysis of 81 genes from 64 plastid genomes resolves relationships in angiosperms and identifies genome-scale evolutionary patterns. *Proc. Natl. Acad. Sci. U.S.A.* 104 19369–19374. 18048330 10.1073/pnas.0709121104 PMC2148296

[B24] Katoh K. Standley D. M. (2013). MAFFT multiple sequence alignment software version 7: improvements in performance and usability. *Mol. Biol. Evol.* 30 772–780. 10.1093/molbev/mst010 23329690 PMC3603318

[B25] Lawrence G. H. M. (1951). *Taxonomy of Vascular Plants.* New York: Macmillan.

[B26] Li H. L. (1944). New or noteworthy plants from Southwestern China. *J. Arnold Arbor.* 25:316.

[B27] Li H. L. (1947). Relationship and taxonomy of genus *Brandisia*. *J. Arnold Arbor.* 28 127–136.

[B28] Lin T.-P. Wang Y.-S. (1991). *Paulownia taiwaniana*, a hybrid between *P. fortunei* and *P. kawakamii* (Scrophulariaceae). *Plant Syst. Evol.* 178 259–269.

[B29] Lohse M. Drechsel O. Kahlau S. Bock R. (2013). Organellar Genome DRAW- a suite of tools for generating physical maps of plastid and mitochondrial genomes and visualizing expression data sets. *Nucleic Acids Res.* 41 W575–W581. 23609545 10.1093/nar/gkt289 PMC3692101

[B30] Lu L. Wang H. Blackmore S. Li D.-Z. Dong L.-N. (2007). Pollen morphology of the tribe Rhinantheae (Orobanchaceae) and its systematic significances. *Plant Syst. Evol.* 268 177–198.

[B31] Maheshwari J. K. (1961). The genus *Wightia* Wall. in India with a discussion on its systematic position. *Bull. Bot. Surv. India* 3 31–35.

[B32] McNeal J. R. Bennett J. R. Wolfe A. D. Mathews S. (2013). Phylogeny and origins of holoparasitism in Orobanchaceae. *Am. J. Bot.* 100 971–983. 10.3732/ajb.1200448 23608647

[B33] Nakai T. (1949). Classes, ordinae, familiae, subfamilieae, tribus, genera nova quae attinentad plantas Koreanas. *J. Jap. Bot.* 24 8–14.

[B34] Olmstead R. G. dePamphilis C. W. Wolfe A. D. Young N. D. Elisons W. J. Reeves P. A. (2001). Disintegration of the Scrophulariaceae. *Am. J. Bot.* 88 348–361. 11222255

[B35] Olmstead R. G. Reeves P. A. (1995). Evidence for the polyphyly of the Scrophulariaceae based on chloroplast *rbcL* and *ndhF* sequences. *Ann. Mo. Bot. Gard.* 82 176–193.

[B36] Oxelman B. Kornhall P. Olmstead R. G. Bremer B. (2005). Further disintegration of Scrophulariaceae. *Taxon* 54 411–425. 11222255

[B37] Pennell F. W. (1920). Scrophulariaceae of the south-eastern United States. *Proc. Acad. Nat. Sci. Philad.* 71 224–291. 10.3109/17482960903273056 19929736

[B38] Rebernig C. A. Weber A. (2007). Diversity, development and systematic significance of seed pedestals in Scrophulariaceae (s.l.). *Bot. Jahrb. Syst.* 127 133–150.

[B39] Refulio-Rodriguez N. F. Olmstead R. G. (2014). Phylogeny of lamiidae. *Am. J. Bot.* 101 287–299. 24509797 10.3732/ajb.1300394

[B40] Ren Y.-Q. Deng G.-Y. Li X.-P. Marczewski T. Ma Y.-P. (2018). Secondary pollen presentation on anther hairs enhances reproductive fitness in *Brandisia hancei*, a protogynous perennial with autonomous selfing. *Plant Ecol. Divers.* 11 373–381.

[B41] Reveal J. L. (2011). Summary of recent systems of angiosperm classification. *Kew Bull.* 66 5–48.

[B42] Ronquist F. Teslenko M. van der Mark P. Ayres D. L. Darling A. Höhna S. ( (2012). MrBayes 3.2: efficient bayesian phylogenetic inference and model choice across a large model space. *Syst. Biol.* 61 539–542. 10.1093/sysbio/sys029 22357727 PMC3329765

[B43] Schäferhoff B. Fleischmann A. Fischer E. Albach D. C. Borsch T. Heubl G. (2010). Towards resolving Lamiales relationships: insights from rapidly evolving chloroplast sequences. *BMC Evol. Biol.* 10:352. 10.1186/1471-2148-10-352 21073690 PMC2992528

[B44] Stamatakis A. (2014). RAxML version 8: a tool for phylogenetic analysis and post-analysis of large phylogenies. *Bioinformatics* 30 1312–1313. 10.1093/bioinformatics/btu033 24451623 PMC3998144

[B45] Stevens P. F. (2001). *Angiosperm Phylogeny Website. Version. 14, July 2017.*

[B46] Takhtajan A. L. (1980). Outline of the classification of flowering plants (Magnoliophyta). *Bot. Rev.* 46 225–359.

[B47] Takhtajan A. L. (1997). *Diversity and Classification of Flowering Plants.* New York, NY: Columbia University Press.

[B48] Tank D. C. Beardsley P. M. Kelchner S. A. Olmstead R. G. (2006). Review of the systematics of Scrophulariaceae s.l. and their current disposition. *Aust. Syst. Bot.* 19 289–307.

[B49] Tsoong P.-C. (1979). “Paulownia,” in *Flora Reipublicae Popularis Sinicae, Vol. 67, Part 2, Scrophulariaceae*, eds Tsoong P. C. Yang H. P. (Beijing: Science Press), 17–46.

[B50] van Steenis C. G. G. J. (1949). Notes on the genus *Wightia* (Scrophulariaceae). *Bull. Bot. Gard. Buitenzorg.* 18 213–227. 10.11646/zootaxa.3734.1.1 25277890

[B51] Wallich N. (1830). *Plantae Asiaticae Rariores*, Vol. 1 London: Treuttel and Wurtz.

[B52] Wang W.-Y. Pai R.-C. Lai C.-C. Lin T.-P. (1994). Molecular evidence for the hybrid origin of *Paulownia taiwaniana* based on RAPD markers and RFLP of chloroplast DNA. *Theor. Appl. Genet.* 89 271–275. 10.1007/BF00225153 24177840

[B53] Wei Z.-X. (1989). Pollen morphology of *Wightia* and its taxonomic significance. *Acta Bot. Yunnan.* 1 65–70.

[B54] Wen J. Harris A. J. Kalburgi Y. Zhang N. Xu Y. Zheng W. (2018). Chloroplast phylogenomics of the New World grape species (*Vitis*, Vitaceae). *J. Syst. Evol.* 56 297–308. 10.1016/j.ympev.2011.11.015 22138159

[B55] Wettstein R. (1891). “Scrophulariaceae,” in *Die Natürlichen Pflanzenfamilien IV/3B*, eds Engler A. Prantl K. (Leipzig: Engelmann), 39–107.

[B56] Willis J. C. (1955). *A Dictionary of the Flowering Plants and Ferns.* Cambridge: Cambridge University Press.

[B57] Xia Z. Wang Y.-Z. Smith J. F. (2009). Familial placement and relations of *Rehmannia* and *Triaenophora* (Scrophulariaceae s. l.) inferred from five gene regions. *Am. J. Bot.* 96 519–530. 10.3732/ajb.0800195 21628207

[B58] Xia Z. Wen J. (2018). The complete chloroplast genome of the endangered species *Triaenophora shennongjiaensis* (Orobanchaceae s.l.). *Mitochondr. DNA B* 3 506–507. 10.1080/23802359.2018.1467242 PMC7799507 33474222

[B59] Xu C. Dong W.-P. Li W.-Q. Lu Y.-Z. Xie X.-M. Jin X.-B. (2017). Comparative analysis of six *Lagerstroemia* complete chloroplast genomes. *Front. Plant Sci.* 8:15. 10.3389/fpls.2017.00015 28154574 PMC5243828

[B60] Yu W.-B. Randle C.-P. Lu L. Wang H. Yang J.-B. dePamphilis C. W. (2018). The hemiparasitic plant *Phtheirospermum* (Orobanchaceae) is polyphyletic and contains cryptic species in the Hengduan Mountains of Southwest China. *Front. Plant Sci.* 9:142. 10.3389/fpls.2018.00142 29479366 PMC5812252

[B61] Zhang N. Wen J. Zimmer E. A. (2015). Congruent deep relationships in the grape family (Vitaceae) based on sequences of chloroplast genomes and mitochondrial genes via genome skimming. *PLoS One* 10:e0144701. 10.1371/journal.pone.0144701 26656830 PMC4682771

[B62] Zhang Z.-Y. (1990). “Orobanchaceae,” in *Flora Reipublicae Popularis Sinicae*, Vol. 69 ed. Wang W. T. (Beijing: Science Press), 69–124.

[B63] Zhou Q.-M. Jensen S.-R. Liu G.-L. Wang S. Li H.-Q. (2014). Familial placement of *Wightia* (Lamiales). *Plant Syst. Evol.* 300 2009–2017.

[B64] Zimmer E. A. Wen J. (2015). Using nuclear gene data for plant phylogenetics: progress and prospects II. Next-gen approaches. *J. Syst. Evol.* 53 371–379.

