# Supplementary figures and images for: Does the Enigmatic Wightia Belong to Paulowniaceae (Lamiales)?
Source: Front Plant Sci. 2019 Apr 30;10:528. doi: 10.3389/fpls.2019.00528 (PMC6503002; doi:10.3389/fpls.2019.00528)

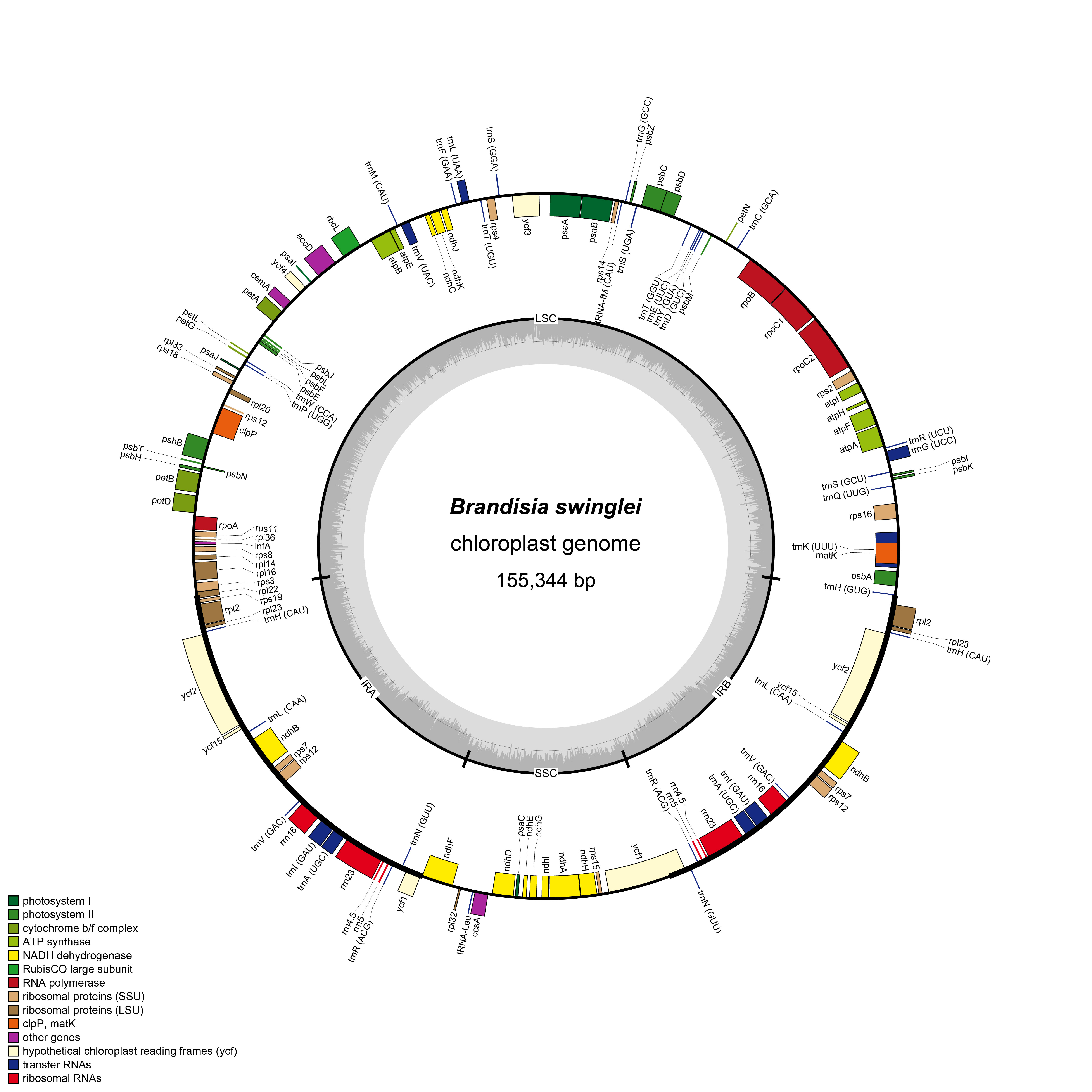

Supplement: FIGURE S1 — Gene map of the chloroplast genome of Brandisia swinglei. [file Image_1.JPEG]

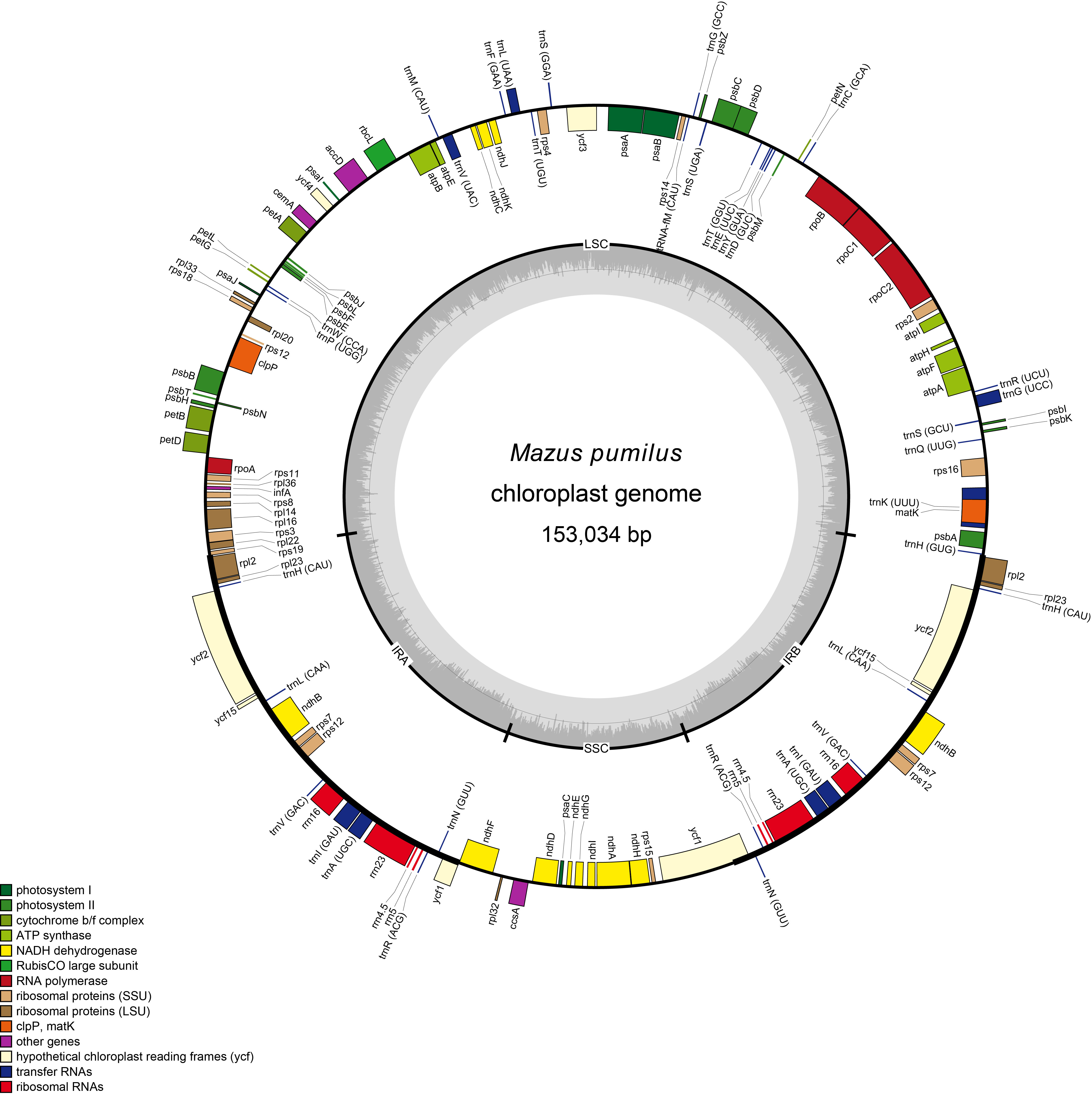

Supplement: FIGURE S2 — Gene map of the chloroplast genome of Mazus pumilus. [file Image_2.JPEG]

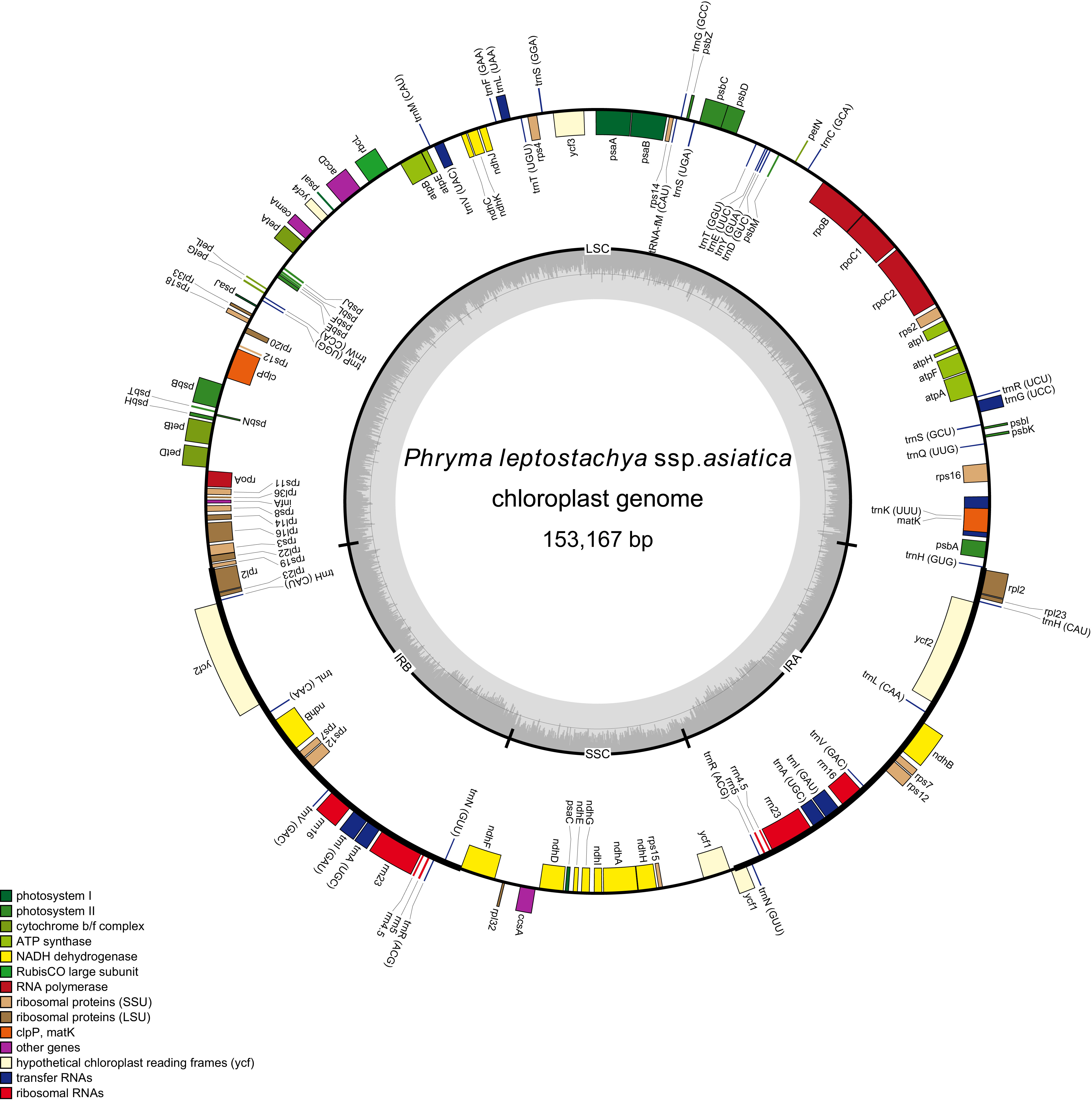

Supplement: FIGURE S3 — Gene map of the chloroplast genome of Phryma leptostachya subsp. asiatica. [file Image_3.JPEG]

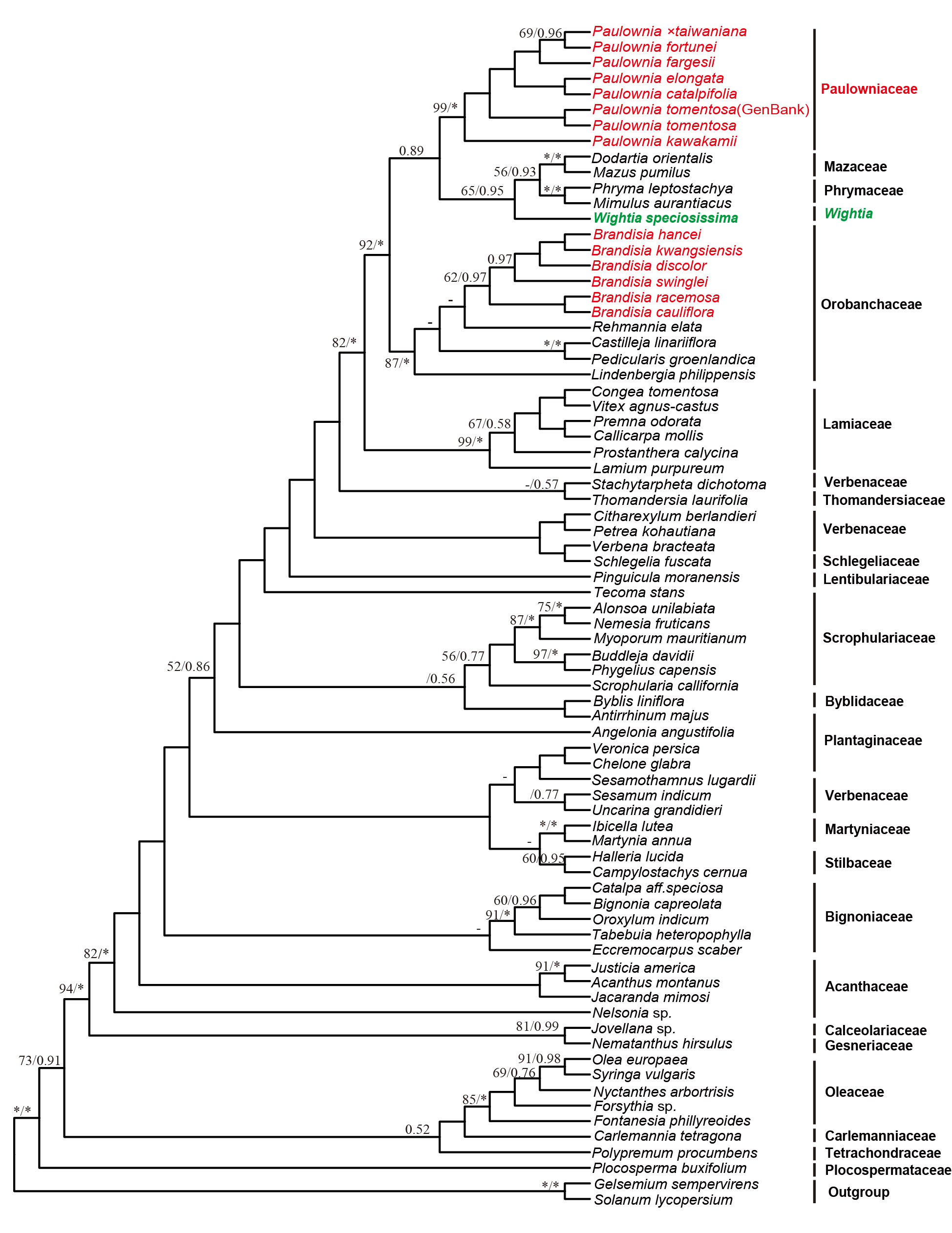

Supplement: FIGURE S4 — Bayesian tree inferred from the sequence of one mitochondrial gene rps3 data. ML bootstrap values (>50%)/Bayesian posterior probabilities (>0.50) are shown above branches. An asterisk indicates bootstrap value of 100 or posterior probability of 1.00; a hyphen (-) indicates the branch was not obtained in the ML bootstrap consensus. [file Image_4.JPEG]
