# Supplementary material for: Does the Enigmatic Wightia Belong to Paulowniaceae (Lamiales)?
Source: Front Plant Sci. 2019 Apr 30;10:528. doi: 10.3389/fpls.2019.00528 (PMC6503002; doi:10.3389/fpls.2019.00528)
Supplement: TABLE S3 — The nine chloroplast DNA regions and one mitochondrial gene used in the phylogenetic analyses of Lamiales. [file Table_3.DOCX]

Table S3 The nine chloroplast DNA regions and one mitochondrial gene used in the phylogenetic analyses of Lamiales.

| Gene | Length of alignment | Variable characters | Parsimony informative characters | PIC percentage (%) |
| --- | --- | --- | --- | --- |
| *atpB* | 1430 | 230 | 340 | 23.78 |
| *matK,* | 2023 | 483 | 898 | 44.39 |
| *ndhF* | 2374 | 475 | 792 | 33.36 |
| *psbBTNH* | 2377 | 329 | 549 | 23.10 |
| *rbcL* | 1314 | 200 | 269 | 20.47 |
| *rps4* | 525 | 91 | 148 | 28.19 |
| *rps16* | 1321 | 302 | 437 | 33.09 |
| *trnL-F* | 1373 | 270 | 452 | 32.92 |
| *trnV-atpE* | 2052 | 539 | 571 | 27.83 |
| *rps3* | 1561 | 333 | 164 | 10.51 |
