# Supplementary material for: Does the Enigmatic Wightia Belong to Paulowniaceae (Lamiales)?
Source: Front Plant Sci. 2019 Apr 30;10:528. doi: 10.3389/fpls.2019.00528 (PMC6503002; doi:10.3389/fpls.2019.00528)
Supplement: TABLE S4 — The 79 protein-coding plastid genes used in the phylogenetic analyses of Lamiales. [file Table_4.DOCX]

Table S4 The 79 protein-coding plastid genes used in the phylogenetic analyses of Lamiales.

| Gene | Length of alignment | Variable characters | Parsimony informative characters | PIC percentage (%) |
| --- | --- | --- | --- | --- |
| *rps12* | 424 | 42 | 42 | 9.90 |
| *ndhB* | 1,755 | 185 | 75 | 4.27 |
| *rpl2* | 864 | 123 | 106 | 12.27 |
| *rpl23* | 294 | 30 | 13 | 5.20 |
| *rps7* | 471 | 57 | 68 | 14.43 |
| *ycf2* | 8429 | 1825 | 1082 | 12.88 |
| *petL* | 96 | 13 | 23 | 23.96 |
| *psbN* | 132 | 12 | 15 | 11.37 |
| *atpH* | 246 | 19 | 39 | 15.85 |
| *atpB* | 1499 | 166 | 307 | 20.48 |
| *atpE* | 421 | 62 | 107 | 25.42 |
| *atpF* | 624 | 86 | 143 | 22.92 |
| *atpI* | 747 | 87 | 149 | 19.95 |
| *ccxA* | 1009 | 156 | 328 | 32.50 |
| *cemA* | 711 | 148 | 196 | 27.57 |
| *clpP* | 674 | 177 | 255 | 37.83 |
| *infA* | 252 | 51 | 81 | 32.14 |
| *matK* | 1703 | 330 | 756 | 44.39 |
| *ndhA* | 1104 | 139 | 262 | 23.73 |
| *ndhC* | 363 | 45 | 63 | 17.36 |
| *ndhD* | 1519 | 233 | 392 | 25.80 |
| *ndhE* | 306 | 59 | 67 | 21.90 |
| *ndhF* | 2352 | 413 | 752 | 31.97 |
| *ndhG* | 537 | 76 | 111 | 20.67 |
| *ndhH* | 1202 | 146 | 249 | 20.71 |
| *ndhI* | 509 | 67 | 112 | 22.00 |
| *ndhJ* | 477 | 69 | 80 | 16.77 |
| *ndhK* | 705 | 82 | 147 | 20.85 |
| *petA* | 969 | 112 | 228 | 23.53 |
| *petB* | 648 | 42 | 101 | 15.59 |
| *petD* | 531 | 38 | 79 | 14.88 |
| *petG* | 114 | 9 | 15 | 13.16 |
| *petN* | 102 | 14 | 9 | 8.82 |
| *psaA* | 2290 | 358 | 344 | 15.02 |
| *psaB* | 2270 | 308 | 326 | 14.36 |
| *psaC* | 246 | 17 | 42 | 17.07 |
| *psaI* | 111 | 18 | 24 | 21.62 |
| *psaJ* | 137 | 24 | 21 | 15.33 |
| *psbA* | 1135 | 183 | 147 | 12.95 |
| *psbB* | 1529 | 129 | 285 | 18.64 |
| *psbC* | 1442 | 137 | 222 | 15.40 |
| *psbD* | 1071 | 103 | 128 | 11.95 |
| *psbE* | 252 | 35 | 38 | 15.08 |
| *psbF* | 120 | 7 | 12 | 10.00 |
| *psbH* | 229 | 28 | 50 | 21.83 |
| *psbI* | 111 | 26 | 21 | 18.92 |
| *psbJ* | 123 | 17 | 12 | 9.76 |
| *psbK* | 195 | 42 | 36 | 18.46 |
| *psbL* | 117 | 10 | 11 | 9.40 |
| *psbM* | 105 | 10 | 15 | 14.29 |
| *psbT* | 102 | 9 | 16 | 15.69 |
| *psbZ* | 189 | 40 | 30 | 15.87 |
| *accD* | 3026 | 950 | 761 | 25.15 |
| *atpA* | 1,572 | 280 | 351 | 22.33 |
| *rbcL* | 1447 | 139 | 263 | 18.18 |
| *rpl14* | 375 | 61 | 107 | 28.53 |
| *rpl16* | 420 | 67 | 108 | 25.71 |
| *rpl20* | 402 | 70 | 171 | 42.54 |
| *rpl22* | 510 | 85 | 217 | 42.55 |
| *rpl32* | 179 | 38 | 72 | 40.22 |
| *rpl33* | 228 | 39 | 66 | 28.95 |
| *rpl36* | 114 | 14 | 37 | 32.46 |
| *rpoA* | 1040 | 194 | 292 | 28.08 |
| *rpoB* | 3340 | 529 | 685 | 20.51 |
| *rpoC1* | 2280 | 394 | 475 | 20.83 |
| *rpoC2* | 4471 | 790 | 1228 | 27.47 |
| *rps2* | 739 | 119 | 235 | 31.80 |
| *rps3* | 720 | 111 | 265 | 36.81 |
| *rps4* | 628 | 113 | 174 | 27.71 |
| *rps8* | 411 | 73 | 155 | 37.71 |
| *rps11* | 478 | 90 | 174 | 36.40 |
| *rps14* | 303 | 56 | 77 | 25.41 |
| *rps15* | 287 | 50 | 111 | 38.68 |
| *rps16* | 273 | 53 | 66 | 24.18 |
| *rps18* | 431 | 84 | 117 | 27.15 |
| *rps19* | 285 | 58 | 100 | 35.09 |
| *ycf3* | 529 | 53 | 75 | 14.18 |
| *ycf4* | 589 | 106 | 140 | 23.77 |
| *ycf15* | 266 | 44 | 15 | 5.64 |
